# Rectal cancer following radiotherapy for prostate cancer: A propensity‐matched analysis

**DOI:** 10.1111/codi.70365

**Published:** 2026-01-28

**Authors:** M. Goldenshluger, M. A. Abbas, M. Belkovsky, A. Alipouriani, K. Erozkan, G. Alon, M. A. Valente, S. R. Steele, S. D. Holubar, D. Liska, E. Gorgun

**Affiliations:** ^1^ Department of Colorectal Surgery, Digestive Disease & Surgery Institute Cleveland Clinic Cleveland Ohio USA; ^2^ Department of Surgery Al‐Zahra Hospital Dubai UAE

**Keywords:** low anterior resection, oncologic outcomes, pelvic radiotherapy, propensity score matching, prostate cancer, radiation‐induced malignancy, rectal cancer, secondary colorectal cancer

## Abstract

**Aim:**

Patients who have previously received radiation therapy for primary prostate cancer (PPC) face an elevated risk of developing secondary rectal cancer (SRC). However, the clinical presentation, surgical outcomes, and oncological results of SRC in this context remain poorly characterized.

This study aims to compare the clinical and pathological features, as well as treatment outcomes, of patients with primary rectal cancer (PRC) and those with SRC following radiation for prostate cancer.

**Methods:**

Retrospective cohort study using univariate and propensity‐matched analyses.

Data extracted from electronic medical records at a single tertiary institution [2001–2021].

Male patients with rectal cancer (RC) who underwent oncological resection with or without a prior history of prostate cancer radiation. Patients with a <3‐year interval between radiotherapy and RC diagnosis were excluded. The main outcome measures were pathological analysis, postoperative complications and overall survival.

**Results:**

Out of 1,755 patients with RC, 50 cases (2.9%) had SRC. Forty‐three out of the 50 patients were included in the analysis. The median time from radiotherapy to SRC diagnosis was 8 ± 4 years (IQR). Patients with SRC were older, with a mean age of 73.7 ± 8.5 versus 61.1 ± 13 years in the control group (*p* < 0.001), and a higher American Society of Anaesthesiologists (ASA) score (*p* = 0.006). Most SRCs were distal with a median distance from the anal verge of 4.25 cm (IQR 9.5 cm). Only seven patients (16.3%) in the SRC group received neoadjuvant radiation therapy versus 764 (44.8%) of PRC (*p* = 0.001). SRC patients required more extensive surgical interventions, including abdominoperineal resection (46.5% vs. 29.9%), pelvic exenteration (4.7% vs. 0.4%), and fewer sphincter‐preserving procedures, including low anterior resection (48.8% vs. 68.2%) and transanal resection (0% vs. 1.5%) (*p* = 0.02). Propensity score matching with a 1:2 ratio matching for age, body mass index (BMI), ASA score, type of surgery, and pathological staging revealed no differences between the groups regarding tumour differentiation, staging, or postoperative complications. Survival analysis at 6 years showed no significant difference in overall survival between the SRC (53.2%, 95% CI: 35%–71%) and PRC (50.3%, 95% CI: 36%–64%) groups (*p* = 0.61).

**Limitations:**

Retrospective design and reliance on electronic medical records from a single institution.

**Conclusion:**

Patients with PPC developed SRC up to 10 years after radiation therapy. Patients with SRC were typically older with more comorbidities. Fewer patients with SRC underwent neoadjuvant therapy, and as a group, required more extensive surgeries with a lower rate of sphincter preservation compared to patients with PRC. Despite these differences, patients with SRC had similar pathological outcomes and overall survival compared to patients with PRC.

**What does this paper add to the literature?**

This study provides comprehensive surgical and oncologic outcomes for secondary rectal cancer after prostate radiotherapy using a propensity‐matched cohort. Patients with secondary rectal cancer were older, had more comorbidities, and most tumors were located in the distal and anterior rectum. Despite more complex surgery, lower sphincter preservation, and limited neoadjuvant options, patients achieved comparable pathologic and survival outcomes to primary rectal cancer. These findings challenge assumptions of worse prognosis and support aggressive surgical management.

## INTRODUCTION

Secondary rectal cancer (SRC) following pelvic radiation therapy (RT) for primary prostate cancer (PPC) presents a unique clinical challenge, necessitating an understanding of its presentation and treatment outcomes. While previous research has highlighted an association between pelvic RT and the development of SRC, there remains a lack of comprehensive data describing short and long‐term outcomes [1].

The utilization of RT, encompassing both external beam radiation (EBRT) and brachytherapy (BT), is an integral component of PPC treatment [2]. The pathophysiological mechanism of RT involves damage to cancer cell DNA, either through direct exposure or the creation of free radicals [3]. This inhibits the replication of cancer cells and initiates cellular reactions that eventually culminate in cell death. In general, individuals who have experienced an initial malignant tumour are more prone to developing a subsequent malignancy compared to the general population [4, 5]. Furthermore, the utilization of RT may contribute to an increased risk of carcinogenesis due to the late effects of DNA damage [1]. Typically, the risk for secondary cancers in patients with prior RT is observed in organs located within or near the irradiated area [6]. Notably, a recent study demonstrated that patients with colorectal cancer (CRC) occurring as a second primary malignancy following another cancer experience poorer survival outcomes compared to patients with primary CRC not preceded by another malignancy [7]. RT associated SRC can occur in the setting of various types of pelvic malignancies in both males and females, with several studies reporting this association in men with PPC [1, 8, 9, 10]. To date, there is limited data on the presentation and treatment outcome of patients with SRC following RT for PPC. Some studies have raised concerns that SCR following RT for PPC presents more often in the distal rectum and on the anterior wall adjacent to the treated prostate [2, 8, 9, 11]. The optimal treatment, type of surgical interventions, and oncological outcomes remain largely unexplored.

This study aimed to compare the clinical presentation, treatment interventions, and outcomes of patients diagnosed with SRC after RT for PPC with cases of primary rectal malignancy without prior RT.

## METHODS

**FIGURE 1:**
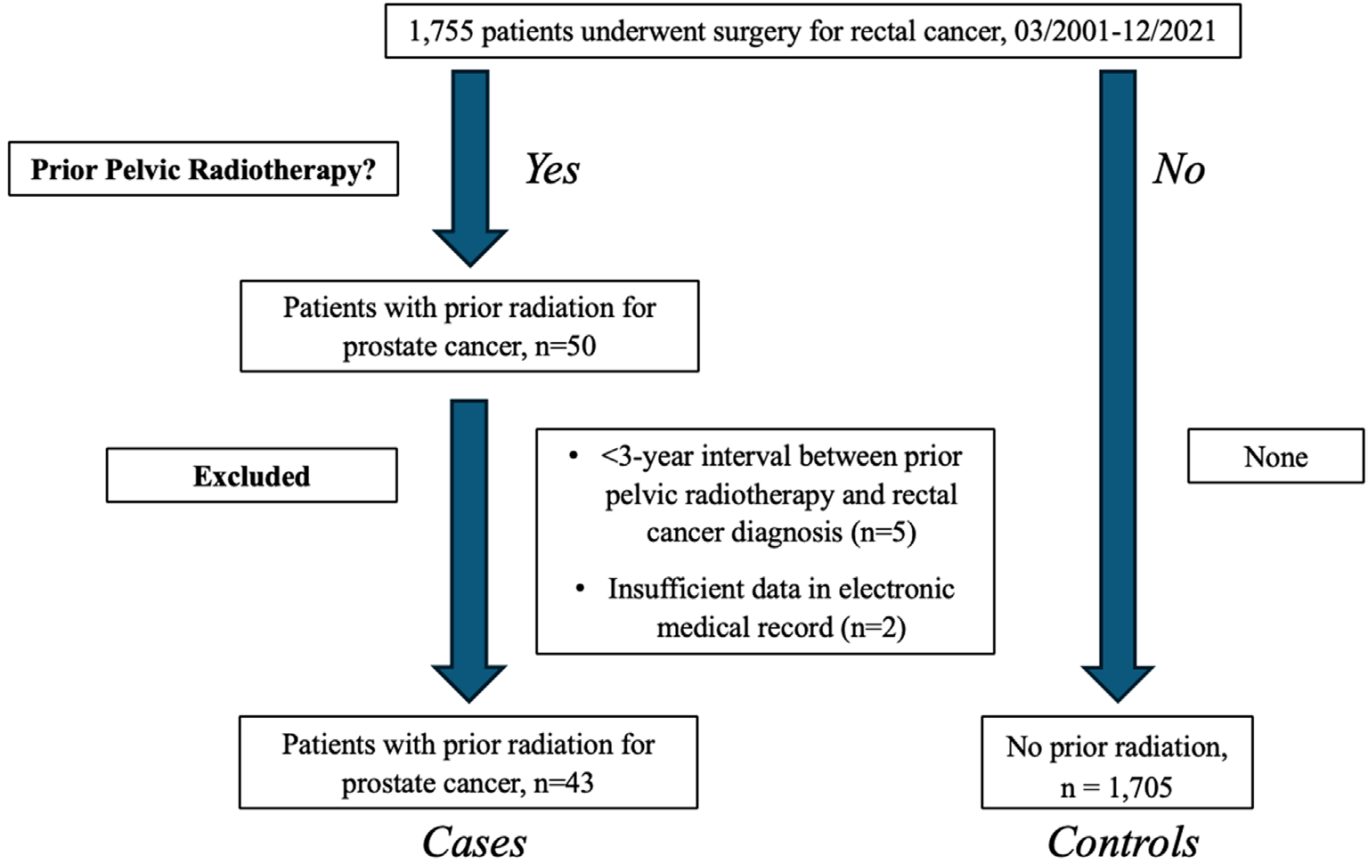
CONSORT‐style Flow Chart of patients with rectal cancer with and without previous pelvic radiotherapy (*n* = 1,755). CONSORT, Consolidated Standards of Reporting Trials.

We retrospectively analysed male patients diagnosed with RC who underwent curative surgical resection at the Cleveland Clinic [Cleveland, Ohio] between March 2001 and December 2021. Data was abstracted from the electronic medical record (EMR). Patients with less than a 3‐year interval between RT and RC diagnosis were excluded to prevent confounding factors that might affect the outcomes and survival analysis (as shown in the CONSORT chart in Figure 1). Patients were divided into two groups: Group 1, those with a history of pelvic RT for PPC, including EBRT and BT, and Group 2, those without. Propensity score matching (PSM) was performed to reduce potential confounding by balancing key baseline characteristics between groups. The variables included in the matching model—age, body mass index (BMI), American Society of Anaesthesiologists (ASA) score, surgery type, and pathological staging—were selected a priori based on clinical relevance and known association with outcomes. A 2:1 matching ratio was used, employing the “optimal” matching method without replacement. The matching was conducted using the Average Treatment Effect for the Treated (ATT) framework. A calliper width of 0.2 standard deviations of the logit of the propensity score was applied to ensure adequate matching quality. Covariate balance after matching was assessed using standardized mean differences (SMDs), with values below 0.1 considered indicative of acceptable balance. Post‐matching comparisons between groups were conducted using Fisher's exact test or chi‐squared test for categorical variables, and the Wilcoxon rank‐sum test for continuous variables, as appropriate.

In addition to the propensity score–matched analysis already presented, we also performed a cohort multivariable logistic regression model adjusting for age, BMI, ASA class, tumour height, tumour grade, lymphovascular invasion, neoadjuvant radiotherapy, neoadjuvant chemotherapy, and surgery type. All statistical analyses were performed using R, version 4.3.1. (www.r‐project.org).

This retrospective study utilized data extracted from electronic medical records; accordingly, a waiver of informed consent was granted by the institutional review board.

## RESULTS

A total of 1,755 patients underwent surgery for RC. Among them, 50 patients (Group 1) had prior pelvic RT for PPC, accounting for 2.9% of the total. The remaining 1,705 patients formed the control group (Group 2).

The baseline characteristics of the participants are shown in Table 1. Seven patients in Group 1 were excluded: five patients with <3‐year duration between RT and RC diagnosis and two due to incomplete data in the electronic medical record (Figure 1). Ultimately, 43 patients (2.5%) were included in Group 1. Among them, 26 patients (60.5%) received EBRT for a total dose of 70 grey (Gy), 12 patients (27.9%) underwent BT with Iodine‐125 (I‐125) seed implantation, and 5 patients (11.6%) received both EBRT and BT. Furthermore, eight patients (18.6%) received EBRT as salvage therapy to the prostatic fossa due to rising prostate‐specific antigen (PSA) levels following prostatectomy, in addition to the primary EBRT dose.

**TABLE 1:** Baseline characteristics of patients with rectal cancer with and without previous pelvic radiotherapy (*n* = 1,748).

Variable	Rectal cancer		*p*‐value
	Primary RC, *n* = 1,705 (97.5%)	Prior RT and SRC, *n* = 43 (2.5%)	
Age, years	61.1 (±13.0)	73.7 (±8.52)	<0.001
BMI, kg/m^2^	28.0 (±6.4)	27.3 (±3.59)	0.53
ASA			
I	12 (0.7%)	0 (0%)	0.006
II	536 (31.4%)	4 (9.3%)	
III	1,032 (60.5%)	34 (79.1%)	
IV	93 (5.5%)	5 (11.6%)	
Unknown	32 (1.9%)		
Distance from AV, cm	4.86 (±4.99)	4.94 (±5.08)	0.91
Preoperative treatment			
Radiotherapy	764 (44.8%)	7 (16.3%)	<0.001
Chemotherapy	793 (46.5%)	10 (23.3%)	0.004
Type of surgery			
LAR	1163 (68.2%)	21 (48.8%)	0.02
APR	509 (29.9%)	20 (46.5%)	
Pelvic exenteration	7 (0.4%)	2 (4.7%)	
Transanal excision	26 (1.5%)	0 (0%)	

*Note*: Numbers represent frequency (proportion) or mean (standard deviation).

Abbreviations: APR, abdominoperineal resection; ASA, American Society of Anesthesiologists; AV, anal verge; BMI, body mass index; LAR, low anterior resection; RC, rectal cancer; RT, radiotherapy, SRC, secondary rectal cancer.

The median latency period from the completion of RT to RC diagnosis was 8 ± 4 years (IQR). Patients in Group 1 were older, with a mean age of 73.7 years ± 8.5 years compared to 61.1 years ± 13 years in Group 2 (*p* < 0.001). Moreover, patients in Group 1 had a higher ASA score compared to Group 2 (*p* = 0.006).

**FIGURE 2:**
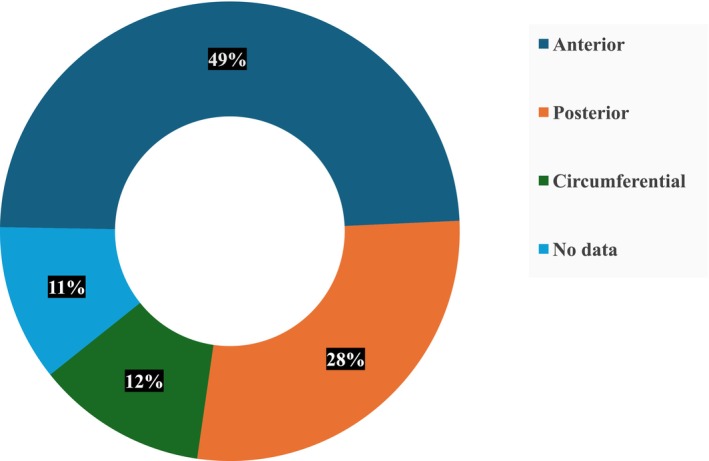
Rectal tumour location in patients with rectal cancer after previous pelvic radiotherapy (*n* = 43).

**FIGURE 3:**
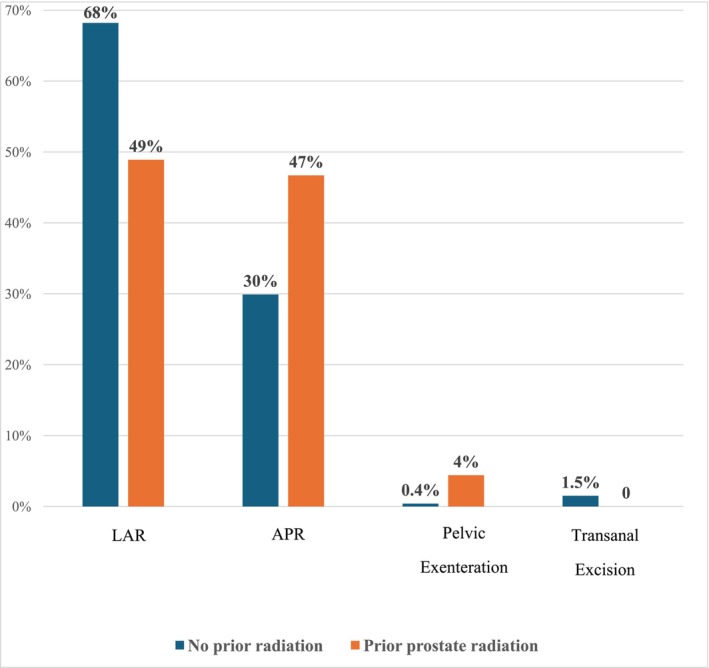
Types of surgeries performed in patients with rectal cancer with and without previous pelvic radiotherapy (*n* = 1,748), *p* = 0.02. APR, abdominoperineal resection; LAR, low anterior resection.

The majority of SRC‐diagnosed Group 1 were in the distal rectum, with a median distance of 4.9.25 cm ± 9.5 cm (IQR) from the anal verge. Of these, 21 patients (48.8%) had tumours located anterolaterally, 12 patients (27.9%) had tumours in a posterior location and 5 patients (11.6%) had circumferential tumours (Figure 2). There were no significant differences between the two groups in terms of tumour differentiation and pathological stage. Only 7 patients (16.3%) in Group 1 received neoadjuvant radiotherapy compared to 764 patients (44.8%) in Group 2 (*p* = 0.01). Patients in Group 1 required more extensive surgical interventions including abdominoperineal resection (46.5% vs. 29.9%) and pelvic exenteration (4.7% vs. 0.4%), and fewer sphincter‐preserving procedures including low anterior resection (48.8% vs. 68.2%) and transanal resection (0% vs. 1.5%), *p* = 0.02 (Figure 3). Histopathological evaluation indicated a higher incidence of radial margin involvement (11.1% vs. 4.3%, *p* = 0.03) and angiolymphatic invasion (26.7% vs. 10.9%, *p* < 0.001) in Group 1. Propensity score matching was conducted with a 1:2 ratio (43 patients in Group 1 and 86 patients in Group 2), matching for age, BMI, ASA score, type of surgery (including anterior resection, abdominoperineal resection (APR), and pelvic exenteration), and pathological stage. A tendency towards earlier T stage was observed in Group 1, with 9 (20.9%) T1 tumours compared to 6 (7%) in Group 2 (*p* = 0.06) (Table 2). No significant differences in early postoperative complications (pelvic abscess, leakage rate and ileus) were noted between the two groups (Table 2).

**TABLE 2:** Results after propensity score matching of patients with rectal cancer with and without previous pelvic radiotherapy (*n* = 129).

Variable	Rectal cancer		*p*‐value
	Prior RT and SRC, *n* = 43 (33.3%)	Primary RC, *n* = 86 (66.7%)	
Age, years	73.5 (±8.68)	73.4 (±10.7)	0.97
BMI, kg/m^2^	27.2 (±3.65)	27.0 (±4.94)	0.56
ASA classification			0.77
II	4 (9.3%)	6 (7%)	
III	33 (76.7%)	64 (74.4%)	
IV	6 (14.0%)	16 (18.6)	
Type of surgery performed			0.13
APR	20 (46.5%)	48 (55.8%)	
LAR	21 (48.8%)	38 (44.2%)	
Pelvic exenteration	2 (4.7%)	0 (0%)	
Operative outcomes			
Operating time, min	244 (±94.6)	237 (±113)	0.40
EBL, ml's	397 (±458)	423 (±671)	0.26
Pathological stage			0.61
1	14 (32.6%)	20 (23.3%)	
2	10 (23.3%)	18 (20.9%)	
3	14 (32.6%)	34 (39.5%)	
4	5 (11.6%)	14 (16.3%)	
Oncological outcomes			
Involved lymph nodes	1.81 (±3.5)	2.91 (±5.7)	0.42
Angiolymphatic invasion	12 (27.9%)	26 (30.2%)	0.40
Radial margins involved	5 (11.6%)	8 (9.3%)	0.31
Distal margins involved	0 (0)	0 (0%)	1.0
Length of stay	10.7 (±7.9)	9.2 (±6.2)	0.28
Complications			
Any postoperative complication	15 (34.9%)	34 (39.5%)	0.61
Intraabdominal abscess	3 (7.0%)	3 (3.5%)	0.34
Pelvic abscess	2 (4.7%)	6 (7.0%)	1.0
Anastomotic leak	2 (4.7%)	3 (3.5%)	0.61
Ileus	5 (11.6%)	11 (12.8%)	0.77

*Note*: Numbers represent frequency (proportion) or mean (standard deviation).

Abbreviations: APR, abdominoperineal resection; ASA, American Society of Anaesthesiologists; BMI, body mass index; EBL, estimated blood loss; LAR, low anterior resection; RC, rectal cancer; RT, radiotherapy; RT, radiotherapy; SRC, secondary rectal cancer.

In multivariable logistic regression models of the full cohort—adjusted for age, BMI, ASA class, tumour height, tumour grade, lymphovascular invasion, neoadjuvant radiotherapy, neoadjuvant chemotherapy, and surgery type—secondary rectal cancer was not independently associated with increased odds of mortality. Adjusted odds ratios with corresponding 95% confidence intervals for all binary outcomes are presented in Table 3.

**TABLE 3:** Adjusted odds ratios for mortality from multivariable logistic regression.

Variable	OR	95% CI	*p*‐value
Previous_radiation for prostate cancer	1.12	0.58–2.19	0.98
Age (per year)	1.05	1.04–1.06	0.82
BMI	1.00	0.98–1.02	0.93
Previous radiation	1.12	0.58–2.19	0.73
Tumour height	1.02	1.00–1.05	0.09
Tumour grade well differentiated	0.97	0.63–1.47	0.88
Tumour grade moderately differentiated	1.02	0.78–1.33	0.90
Tumour grade poorly differentiated	1.16	0.82–1.64	0.40
Lymphovascular invasion	1.54	1.11–2.13	0.009
Preop radiation	0.81	0.47–1.39	0.44
Preop chemotherapy	1.58	0.93–2.72	0.094
Surgery type: LAR	1.02	0.82–1.28	0.83
Surgery type: Pelvic exenteration	2.10	0.65–12.49	0.39

Abbreviations: BMI, body mass index; LAR, low anterior resection; OR, odds ratio.

**FIGURE 4:**
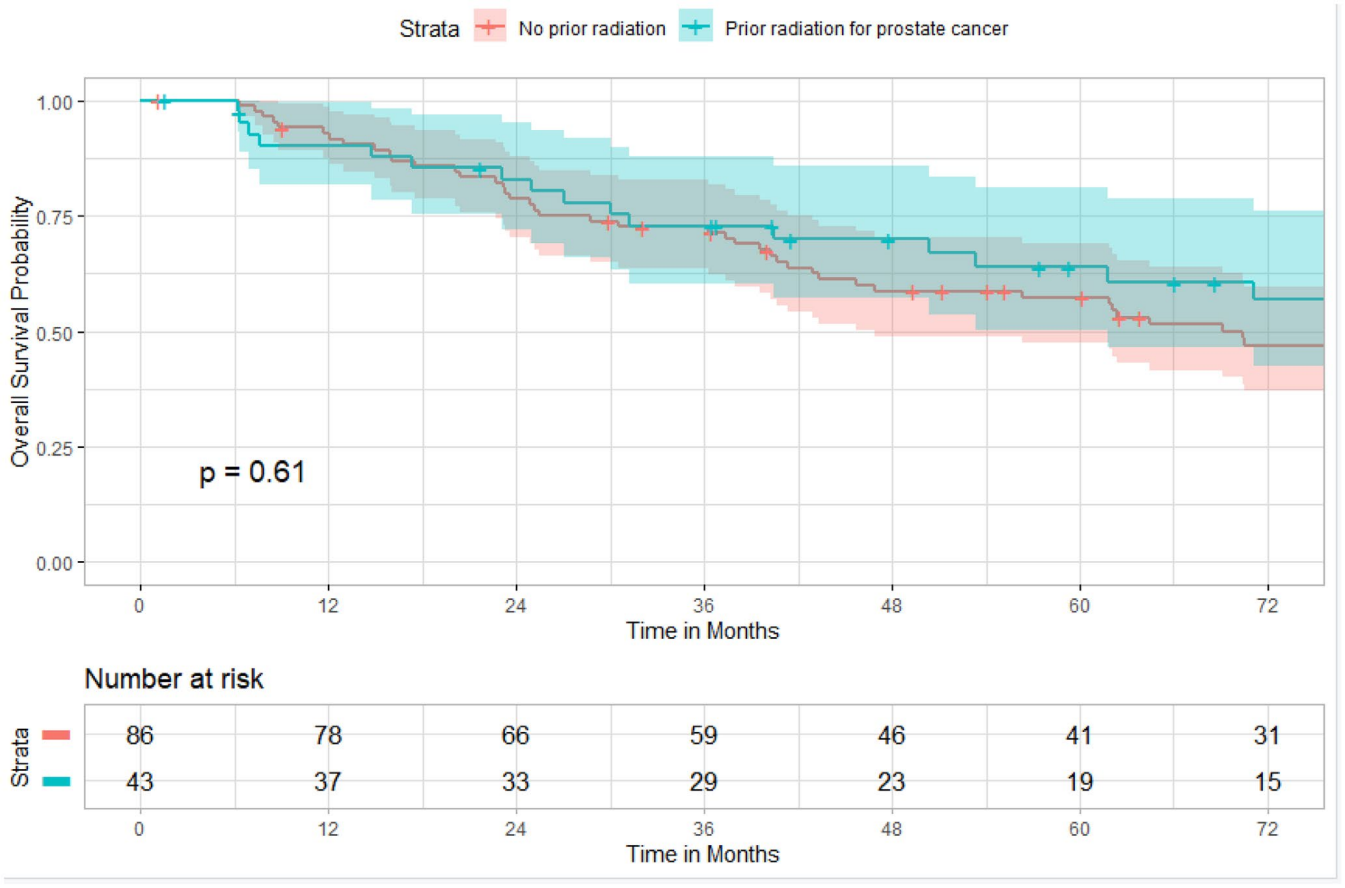
Overall survival probability of matched patients (*n* = 86; 43 per group) with primary versus secondary rectal cancer (*p* = 0.61). At 6 years (72 months): No prior radiation—50.3% (95% CI: 36%–64%) | Prior radiation—53.2% (95% CI: 35%–71%).

Overall survival analysis at 6 years (72 months) showed no significant difference between Group 1 (50.3%) and Group 2 (53.2%), *p* = 0.61 (Figure 4).

## DISCUSSION

Our data underscores several noteworthy observations regarding patients with SRC following RT for PPC. Patients with SRC tended to be older and presented with a higher burden of medical comorbidities. The baseline characteristics of the patient cohort in our study are consistent with previous research on SRC, indicating a trend towards older age, increased comorbidities, in patients with secondary malignancies post‐RT. [1, 11] Furthermore, we noted that most patients with SRC could not receive neoadjuvant radiotherapy due to prior RT exposure, leading to more extensive resections and fewer sphincter‐preserving surgical interventions. Despite these findings, no differences were observed between the two matched groups in terms of tumour differentiation, postoperative complications, or 6‐year overall survival. These findings mirror those reported previously by Rombouts et al. in their retrospective analysis [12]. In our cohort, just under 50% of patients had tumours located in the anterolateral region, potentially challenging the hypothesis that these tumours are predominantly linked to radiation‐induced carcinogenesis. However, an additional 12% of patients had tumours described as circumferential, which cannot be precisely classified by anatomical location. Moreover, tumour location data were unavailable in 11% of cases. Importantly, tumours located strictly in the posterior rectum accounted for less than 30% of the cohort—an observation that remains consistent with patterns typically associated with radiation‐related carcinogenesis [1, 4, 8].

The limited availability of literature on SRC following RT treatment of PPC stems from the challenges associated with its uncommon occurrence and the need for extended follow‐up periods following radiotherapy exposure to the diagnosis of SRC. The appropriate latency time to determine the late pro‐neoplastic effects of ionizing radiation for prostate cancer is still debatable [1]. Hou et al. demonstrated that the standardized incidence ratios of SRC become more significant after 10 years [13]. Other studies have reported SRC occurrence after latency periods ranging from 3 months to 5 years post‐RT. [14, 15] Our analysis only included patients with a minimum follow‐up of 3 years after RT exposure, with a median observed follow‐up of 8 years. We selected a minimum of a 3‐year threshold, taking into consideration a sufficient latency time for radiation‐induced rectal cancer to manifest, while maintaining an adequate sample size for analysis.

The molecular profile pathognomonic of radiotherapy‐related tumours remains to be elucidated, making it difficult to clearly distinguish whether rectal cancer in these patients is radiation‐related [1, 15]. However, a standardized surveillance regimen, including digital rectal examination, endoscopy, and imaging, may facilitate early diagnosis of subsequent SRC cases in patients with a history of prostate cancer treated with RT. Previous studies on the actual incidence of SRC have highlighted the impact of increased screening in this population, potentially leading to earlier detection of rectal cancers [13, 14, 15]. We believe that patients with a history of PPC who have received radiation therapy should undergo colorectal cancer screening, similar to other high‐risk populations [16]. Given that most tumours in this group tend to be low and anterior, annual digital rectal examination and sigmoidoscopy for up to 10 years may be beneficial. This approach could facilitate earlier detection, as our study showed a trend towards lower T‐stage at diagnosis among patients with SCR, which we attribute to the close follow‐up practices for previously irradiated patients at our patients with SCR (*p* = 0.06, Table 4).

**TABLE 4:** Pathologic characteristics of patients with rectal cancer with and without previous pelvic radiotherapy (*n* = 1,748).

Variable	Rectal cancer		*p*‐value
	Prior RT and SRC *n* = 43 (2.5%)	Primary RC *n* = 1,705 (97.5%)	
Tumour differentiation			0.68
Well	4 (9.3%)	129 (7.6%)	
Moderately	19 (44.2%)	784 (46.0%)	
Poorly	6 (13.9%)	185 (10.9%)	
Unknown	14 (32.6%)	607 (35.6%)	
Pathological staging			0.17
1	8 (18.6%)	498 (29.2%)	
2	7 (16.3%)	272 (16.0%)	
3	10 (23.3%)	354 (20.8%)	
4	5 (11.6%)	113 (6.6%)	
Unknown	13 (30.2%)	468 (27.4%)	
Pathological T stage			0.26
Tis	0 (0%)	162 (9.5%)	
T1	3 (6.9%)	126 (7.4%)	
T2	9 (20.9%)	379 (22.2%)	
T3	20 (46.5%)	659 (38.7%)	
T4	2 (4.7%)	62 (3.6%)	
Unknown	9 (20.9%)	317 (18.6%)	
Pathological N stage			0.40
N0	18 (41.9%)	927 (54.4%)	
N1	9 (20.9%)	322 (18.9%)	
N2	7 (16.3%)	183 (10.7%)	
Unknown	9 (20.9)	273 (16%)	
Angiolymphatic invasion	12 (27.9%)	185 (10.9%)	<0.001
Positive margins			
Radial margin	5 (11.6%)	74 (4.3%)	0.03
Distal margin	0 (0%)	9 (0.5%)	1.0
Distance to margins, cm			
Radial margin	1.3 (±1.8)	0.6 (±1.1)	<0.001
Distal margin	3.4 (±3.0)	1.8 (±2.6)	<0.001
Nearest margin	1.7 (±3.9)	1.5 (±2.1)	0.4

*Note*: Numbers represent frequency (proportion) or mean (SD). RC: Rectal Cancer; RT: Radiotherapy, SRC: secondary rectal cancer.

Abbreviations: RC, rectal cancer; RT, radiotherapy; SRC, secondary rectal cancer.

This screening regimen should be followed in any patient who receives pelvic RT, regardless of RT technique. In our study, the SRC group included prior exposure to EBRT, BT, and salvage RT. Past research has suggested that both EBRT and BT may contribute to the development of SRC, with EBRT being associated with a slightly elevated risk [17].

We recognize the limitations of our study, particularly its retrospective design and reliance on data from previous electronic medical records. Environmental factors, such as smoking, which could have influenced cancer incidence, were not accounted for because of the absence of such data in the EMR. Additionally, the lack of sufficient data prevented the analysis of long‐term rectal cancer‐specific survival and recurrences; therefore, only overall survival outcomes are reported here. This limitation is due to the nature of our clinical practice at the Cleveland Clinic, where we care for local, regional, national, and international patients. Thus, some distant patients receive phone or email follow‐up, which precludes a complete oncological data set.

In conclusion, this study highlights the complex clinical challenges associated with rectal cancer as a secondary malignancy after prostate radiation therapy. They are typically older and have more comorbidities. They are often unable to undergo neoadjuvant therapy and require more extensive surgery with fewer sphincter‐preserving options. Nonetheless, after adjusting for age, ASA score, and cancer stage, their overall survival was comparable to that of patients with PRC. Further research using larger databases or multi‐institutional studies is necessary to evaluate this issue in greater depth and to generate cancer‐specific survival and disease‐free survival data using Kaplan–Meier analysis. The ideal colorectal cancer screening protocol for patients with PPC who undergo RT should be standardized at a national level to provide guidance to the treating physicians.

## AUTHOR CONTRIBUTIONS

**M. Goldenshluger:** Conceptualization; investigation; writing – original draft; methodology; validation; formal analysis; project administration; data curation; resources. **M. A. Abbas:** Supervision; writing – review and editing. **M. Belkovsky:** Writing – original draft; formal analysis; data curation; conceptualization. **A. Alipouriani:** Data curation; methodology; writing – review and editing. **K. Erozkan:** Resources; data curation; writing – review and editing. **G. Alon:** Writing – review and editing; data curation; methodology. **M. A. Valente:** Supervision; resources. **S. R. Steele:** Supervision; resources. **S. D. Holubar:** Supervision; writing – review and editing. **D. Liska:** Supervision; conceptualization; methodology. **E. Gorgun:** Conceptualization; methodology; supervision.

## FUNDING INFORMATION

This study received no funding.

## CONFLICT OF INTEREST STATEMENT

The authors declare no conflicts of interest.

## ETHICS STATEMENT

The authors are accountable for all aspects of the study and ensure that questions related to the accuracy or integrity of any part of the study are appropriately investigated and resolved. This study was deemed IRB‐exempt by the Cleveland Clinic IRB.

## DISCLOSURES

Dr Emre Gorgun received consultancy fees from Boston Scientific, DiLumen, Intuitive, and Olympus. Dr Stefan Holubar received consulting fees from Takeda, research funding from the American Society of Colon and Rectal Surgery, and the Crohn's and Colitis Foundation. The other authors have no conflict of interest or financial ties to disclose.

## ORIGINALITY

This study is original, written without generative AI and has not been previously presented or published elsewhere. During the preparation of this work, the author used PaperPal to proofread grammar and punctuation. After using this tool/service, the author(s) reviewed and edited the content as needed and take full responsibility for the content of the publication.

## PRIOR PRESENTATION

This presentation received the Best Poster Award from the American Society of Colon and Rectal Surgeons Meeting held in Baltimore from June 1 to 4, 2024.

## Data Availability

The data that support the findings of this study are available on request from the corresponding author. The data are not publicly available due to privacy or ethical restrictions.
